# NanoConstruct: A web application builder of ellipsoidal nanoparticles for the investigation of their crystal growth, stability, and the calculation of atomistic descriptors

**DOI:** 10.1016/j.csbj.2024.05.039

**Published:** 2024-05-25

**Authors:** Panagiotis D. Kolokathis, Dimitrios Zouraris, Evangelos Voyiatzis, Nikolaos K. Sidiropoulos, Andreas Tsoumanis, Georgia Melagraki, Kaido Tämm, Iseult Lynch, Antreas Afantitis

**Affiliations:** a NovaMechanics MIKE, Piraeus 18545, Greece; b NovaMechanics Ltd, Nicosia 1070, Cyprus; c Entelos Institute, Larnaca 6059, Cyprus; d Division of Physical Sciences and Applications, Hellenic Military Academy, Vari 16672, Greece; e Institute of Chemistry, University of Tartu, Tartu 50090, Estonia; f School of Geography, Earth and Environmental Sciences, University of Birmingham, Birmingham B15 2TT, United Kingdom

**Keywords:** Nanoparticle, Ellipsoidal, Crystal growth, Stability, Descriptor, Energy minimization, Digital construction, Zirconia (ZrO_2_), Automation

## Abstract

NanoConstruct is a state-of-the-art computational tool that enables a) the digital construction of ellipsoidal neutral energy minimized nanoparticles (NPs) in vacuum through its graphical user-friendly interface, and b) the calculation of NPs atomistic descriptors. It allows the user to select NP’s shape and size by inserting its ellipsoidal axes and rotation angle while the NP material is selected by uploading its Crystallography Information File (CIF). To investigate the stability of materials not yet synthesised, NanoConstruct allows the substitution of the chemical elements of an already synthesized material with chemical elements that belong into the same group and neighbouring rows of the periodic table. The process is divided into three stages: 1) digital construction of the unit cell, 2) digital construction of NP using geometry rules and keeping its stoichiometry and 3) energy minimization of the geometrically constructed NP and calculation of its atomistic descriptors. In this study, NanoConstruct was applied for the investigation of the crystal growth of Zirconia (ZrO_2_) NPs when in the rutile form. The most stable configuration and the crystal growth route were identified, showing a preferential direction for the crystal growth of ZrO_2_ in its rutile form. NanoConstruct is freely available through the Enalos Cloud Platform (https://enaloscloud.novamechanics.com/riskgone/nanoconstruct/).

## Graphical Abstract

**Figure:**
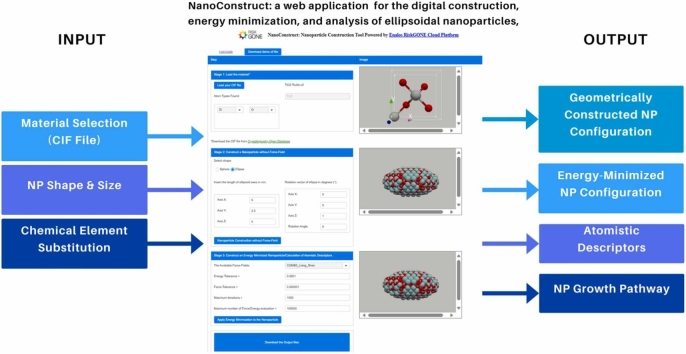

## 1 Introduction

In the continuously evolving landscape of nanotechnology, the design and characterization of nanoparticles (NPs) have garnered significant attention due to their unique properties and potential applications in various fields, including medicine, electronics, and materials science. NPs are crucial in nanotechnology, bridging physics, chemistry, and biology to innovate across multiple sectors. Their unique properties, distinct from their larger counterparts due to increased surface area and quantum effects, enable groundbreaking applications. In medicine, NPs enhance drug delivery, allowing precise targeting of disease cells, reducing side effects, and improving treatment outcomes [1], [2], [3]. In electronics, NPs aid in developing smaller, more efficient devices. Silver and gold NPs, for example, are key in conductive inks for printed electronics, leading to the creation of flexible electronics like wearable sensors [4], [5], [6], [7]. In materials science, NPs like carbon nanotubes contribute to stronger, lighter materials with superior thermal and electrical properties, essential for advanced energy storage and structural applications [8]. Environmental technologies also benefit from NPs, such as titanium dioxide for pollutant breakdown under UV light, pointing towards improved air and water purification methods [9], [10], [11].

A critical aspect of advancing nanotechnology is the theoretical calculation of NPs properties to enable *in silico* screening of the materials that are most useful to be synthesised in the laboratory, in much the same manner as is currently applied in *de novo* drug design [12]. This paper presents a comprehensive study focused on the digital construction and analysis of ellipsoidal NPs using the NanoConstruct web application. NanoConstruct represents a leap forward in computational nanotechnology, offering non-computational scientists a sophisticated platform for the investigation of the crystal growth of NPs without the need for coding skills or deep understanding of the background calculations that is applicable to any NP type. Traditional experimental approaches to NP synthesis and characterisation, and especially to their toxicological evaluation, can be slow, expensive, and lead to non-pure NPs (i.e., NPs consisted of more than one material) with dispersion in their size and shape making it challenging to pinpoint the specific NP features that are responsible for specific functionalities or biological activities [13]. Consequently, a computational tool is needed to investigate the wide range of materials (e.g., there are more than 50,0000 entries in the crystallography open database) in a rapid and efficient manner. NanoConstruct can fill this gap by inserting the Crystallography Information Files (CIFs) of these materials into its Graphical User Interface (GUI) and allowing users to tailor the NPs size, shape, crystal structure and properties systematically.

The number of available materials significantly increases if we consider that NanoConstruct allows the substitution of the chemical elements with others that belong to the same column/group and neighbouring rows/periods of the periodic table which ensures: 1) the absence of overlapping of atoms due to their size which leads NanoConstruct to crash and which cannot occur in real materials, and 2) the maintenance of the chemical bonds of the structure to maintain realistic materials structures. In more detail, elements belonging to the neighbouring rows of periodic table can be replaced due to their similar size while elements belonging to the same group have similar bonding behaviours.

The use of NanoConstruct for investigation of the stability of hypothetical materials enables it to support the development of new materials. The Materials Project [14] (www.materialsproject.org), a core program of the Materials Genome Initiative, also investigates the stability of hypothetical structures but for their bulk phase.NanoConstruct focuses on the NPs stability, which is a stage prior to the bulk phase meaning that it is highly complementary. Energy barriers may make the stable bulk phase predicted by the Materials Project inaccessible and the existence of these barriers can be investigated by NanoConstruct.

To have a better understanding of the capabilities of NanoConstruct, a specific case study has been selected to display the utility of NanoConstruct in materials design and optimisation, and the importance of computational tools in advancing nanotechnology research in general. This case study explores the crystal growth ZrO_2_ NPs when they are in a rutile-like structure (space group P4_2_/mnm).

According to Crystallography Open Database (COD), ZrO_2_ comes in nature in different space groups (e.g., Fm-3 m, P4_2_/nmc, Pbcm, P12_1_/c1) with cubic, tetragonal, orthorhombic, and monoclinic unit cells. However, the structure of ZrO_2_ which is investigated in this paper and belongs to the P4_2_/mnm space group is missing from COD database. This structure corresponds to a tetragonal unit cell, and it can be created from TiO_2_ (rutile) after replacing all Ti atoms with Zr atoms. To the best of our knowledge, there is not a CIF file for this rutile-like structure of ZrO_2_ in literature despite the existence of X-ray Diffraction patterns (XRDs) that reveal its synthesis on the TiO_2_ rutile surface [15] and theoretical works (Materials Project identification number: mp-775909) that predict its bulk stability. The XRDs of Zhang *et al.* [15] are a strong indication that a core of TiO_2_ (rutile) NP can be used for the directed synthesis of ZrO_2_ NPs with a similar structure (space group P4_2_/mnm). The Zr atoms of the rutile-like structure of ZrO_2_ are six coordinated (i.e., forming chemical bonds with six oxygen atoms) in contrast to the Zr atoms of another tetragonal unit cell with space group P4_2_/nmc where Zr atoms are eight-coordinated. We selected to investigate the rutile-like structure of ZrO_2_ to demonstrate the power of NanoConstruct to investigate the stability of materials not yet synthesized based on the structures of already synthesized materials.

Furthermore, ZrO_2_ is a key material in NP research because of its properties and wide use in various fields, important for driving progress in nanotechnology. Its well-known phases are especially valued for their ability to withstand high temperatures and resist corrosion, making it essential for tough industrial uses, including as a catalyst [16]. ZrO_2_ is also chosen for its biocompatibility and strength, making it perfect for dental and medical uses, like crowns, bridges, and implants, due to its durability and safety [17]. Furthermore, ZrO_2_ NPs are explored for catalysis, enhancing reactions, and leading to greener processes [18], [19].

This case study focuses on ZrO_2_ rutile-like NPs, specifically ellipsoid-shaped ones, to examine how its crystal growth can be controlled for different uses.

Studying ZrO_2_ NPs and predicting their crystal growth currently faces challenges, mainly due to the complexity of accurately understanding ZrO_2_ behaviour at the nanoscale. These challenges include modeling interactions within ZrO_2_ crystals and assessing how NP size and shape affect their properties and growth [20], [21]. The NanoConstruct toolbox offers a solution by providing a computational platform for creating and analyzing ZrO_2_ NPs digitally. It calculates the average potential energy per atom for a range of ellipsoids, allowing to identify the growth route by keeping the most stable (low average potential energy per atom) shapes that might be hard to achieve in experiments [22], [23]. This case study demonstrates that NanoConstruct combines precision, customization, efficiency, and cost-effectiveness, accelerating research and reducing costs.

Other works that have attempted the theoretical prediction of bulk phase crystal structures include the investigation of the lattice stability of ReO_3_-type hypothetical TaO_3_ using density functional theory calculations [24], the prediction of new AlF_3_ crystal structures using the GRINSP software, focusing on structures built from AlF_6_ [25], the creation of a comprehensive database of hypothetical zeolite structures using Monte Carlo Methods [26], the prediction of the synthesizability of crystalline materials using color-coded three-dimensional images to represent the atomic structure of crystals, employing convolutional neural networks (CNNs) and convolutional auto-encoders (CAEs) for feature learning and classification [27], [28] as well as the exploration and identification of MOFs with high methane storage capacities via computational screening of hypothetical MOFs [28]. All these hypothetical structures could be inserted into NanoConstruct to investigate their crystal growth and their potential energy barriers.

## 2 Methodology

### 2.1 Digital construction of ellipsoidal NPs

NanoConstruct is divided into three main stages: 1) digital construction of the unit cell, 2) digital construction of NP using geometrical rules and maintaining its stoichiometry and 3) energy minimization of the geometrically constructed NP and calculation of its atomistic descriptors. The importance of calculating atomistic descriptors is pivotal in understanding the complex properties of NPs. These descriptors, both geometrical and atomistic, offer deep insights into the physical and chemical properties of ellipsoidal NPs, facilitating a comparative analysis across a spectrum of materials.

In the first stage, the digital reconstruction of the unit cell of the selected material is carried out by loading its Crystallographic Information File (CIF) into NanoConstruct. An additional feature of NanoConstruct is the option to replace the chemical elements indicated in the CIF file with others from the same group in the periodic table, paving the way for the investigation of hypothetical materials and their properties. The second stage is where the NP geometric digital construction takes place. Here, the unit cell is replicated in all necessary directions to create a simulation box, which forms the foundation for constructing a spherical NP. This process is executed with precision to prevent memory overflow. The geometrical construction of spherical NP is a step that precedes the geometrical construction of the ellipsoid NP while there is an extensive analysis of stage 2 for the geometrical construction of spherical NPs in our previous web application called ASCOT (see Fig. 3 of ASCOT paper) [29].

NanoConstruct is thus an extension of ASCOT [29] as: 1) ASCOT is limited to spherical NPs, while NanoConstruct is extended to enable ellipsoid NPs; 2) NanoConstruct’s coverage of the materials composition landscape is much more inclusive than ASCOT’s due to allowing the selection of any material by uploading its CIF File, while in ASCOT only Ag, CuO and TiO_2_ NPs are accessible, as their CIF files are already uploaded without the need for the user to search for their CIF files in crystallography databases, 3) Force-Fields that lead to crashing of the simulated NPs are excluded from ASCOT while this is not possible for NanoConstruct, as it is intentionally much more general allowing users to explore the whole materials space; and 4) there is no option for chemical substitution in ASCOT, while NanoConstruct allows it to extend the range of materials that can be explored *in silico*.

**Fig. 1:**
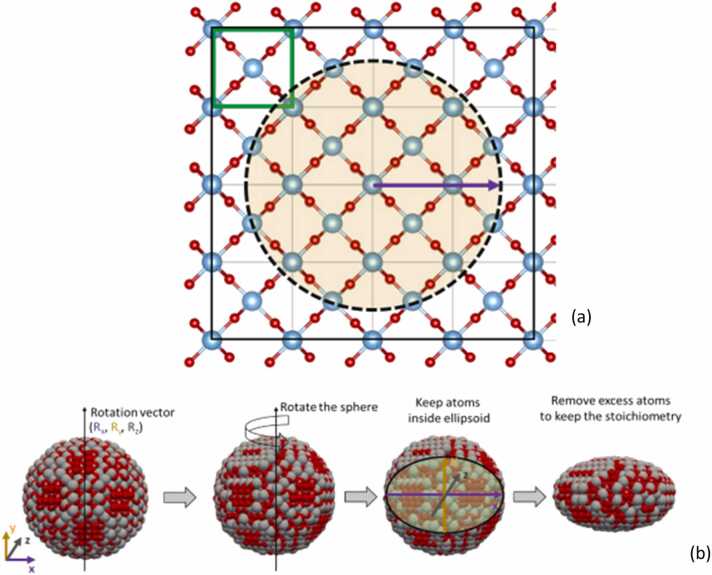
The smallest simulation box (black box) needed for the reconstruction of spherical NP having a specific radius (purple vector), made after the replication of the unit cell (green box) of the rutile phase of TiO_2_ (=four times per direction). The dashed line is the borderline of the sphere that is reconstructed respectively. (b) Procedure of the digital reconstruction of ellipsoidal NPs starting from the spherical NP, rotating the NP around a rotation vector with components Rx, Ry, Rz, keeping the atoms inside the ellipsoid defined by its three ellipsoid axes and finally removing the excess atoms of the ellipsoid to keep the stoichiometry.

To move from the geometrical construction of a spherical NP to the ellipsoid NP, the user defines the shape formation (ellipsoid axes lengths), the rotation angle and rotation axis of the spherical NP (see Fig. 1). The atoms outside the boundaries of a sphere with diameter equal to the maximum axis length of the ellipsoid, are removed. The sphere is then rotated around a user-defined rotation vector. Following this, atoms outside the defined ellipsoidal axes are also removed to achieve the ellipsoid shape. Additionally, to maintain the neutrality of the NP, any excess atoms closest to the ellipsoid's surface are removed, ensuring the correct stoichiometry.

The third and final stage involves energy minimization and the calculation of atomistic descriptors. A list of suitable force-fields is generated, with a preference for the least generic option that still includes all the chemical elements of the NP. The conjugate gradient method is then applied to minimise the energy of the constructed NP, thus enhancing its stability and authenticity. Following this energy minimization, NanoConstruct calculates atomistic descriptors, offering deep insights into the structural and physicochemical properties of the NP.

The aforementioned functions have been grouped into three different Stages, each running independently from the next ones. This signifies that the user does not have to insert the input values of the next Stages, while a separate “Run” button exists for each Stage. Concerning the dependencies of the Stages, each Stage depends on its previous one, meaning that the user should first run Stage 1, next Stage 2 and finally Stage 3 without being able to omit a Stage. However, the NanoConstruct-derived output files can be downloaded after the end of each stage.

### 2.2 Description/definitions of the calculated descriptors

**Fig. 2:**
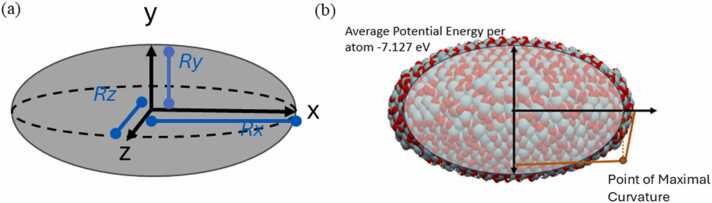
(a) Ellipsoid with its radii a, b and c per principal direction x, y and z respectively. (b) A graph of the average potential energy per atom with a sketch of a nanoparticle Average coordination parameters, CNP and hexatic order parameters for other cutoff values are expected to be added in an updated version of NanoConstruct.

The calculation of atomistic descriptors is key to understand the complex properties of the NPs. During the atomistic descriptor calculations, a uniform surface thickness of 4 Å is employed to distinctly identify the surface and core atoms within the NP, recognizing that NPs have distinct properties in their core and shell regions. This distinction is crucial for ellipsoids, where the surface area is determined by the user-defined ellipsoid and an adjusted ellipsoid, which is derived by reducing the semilengths of its axes by 4 Å, or equivalently, the full lengths by 8 Å (see Fig. 2). The adoption of a consistent measure for surface thickness across all NPs underpins an unbiased comparative analysis of their descriptors, irrespective of their chemical diversity (see ASCOT for more information [29]).

To facilitate a comprehensive analysis, we used the surface thickness to classify NP’s atoms into two distinct categories: core and surface atoms, allowing for the calculation of descriptors for each category individually, as well as for the whole NP. By analyzing the ratios or differences between these descriptors, novel descriptors emerge, offering promising insights into the predictive properties of NPs. Specifically, descriptors formulated as ratios/differences between the core and surface atom descriptors are instrumental in forecasting the reactivity of NPs, with elevated values of these descriptors indicative of a NP's heightened reactivity.

Furthermore, these descriptors are systematically organised into two main types: geometrical and atomistic. This categorization enhances the interpretability and applicability of the descriptors in predicting the physical and chemical properties of NPs. The geometrical construction may differ significantly from the energy-minimised structure (which is the most probable structure for a system in thermal equilibrium [30], underscoring the necessity of the energy minimization procedure before calculating atomistic descriptors. Geometrical are the descriptors which are related to the macroscopic structure of the NP and include the following:

a) Diameters of ellipsoid axis (ellipsoid axes lengths) per x, y, and z direction (the maximum distance between two atoms per direction x, y and z which are notated as *D*x, *D*y and *D*z) (Fig. 2a). These diameters are expected to differ from those inserted by the user because of the structural changes during the energy minimization. Their corresponding ellipsoid axes semilengths are notated as *R*_x_, *R*_y_ and *R*_z_.
b) Surface area of the NP [31] (formula by Knud Thomsen) for the surface of an ellipsoidal NP where *p* ≈ 1.6075 and yields a relative error of at most 1.061 %.
  (1) $$S_{\mathrm{ellipsoid}}=4\pi {\left(\frac{{\left(R_{x}\;R_{y}\right)}^{p}+{\left(R_{x}\;R_{z}\right)}^{p}+{\left(R_{y}\;R_{z}\right)}^{p}}{3}\right)}^{\frac{1}{p}}$$
c) Volume of the ellipsoidal NP

(2) $$V_{\mathrm{ellipsoid}}=\frac{4}{3}\pi R_{x}\;R_{y}\;R_{z}$$

Atomistic are the descriptors which are related to microscopic properties of the NP and include the following:

a) The average potential energy per atom, calculated by the sum of the contribution of each atom to various pairs, bond and/or other potentials of the selected force-field and the division of this by the number of the group of atoms. The contribution of each atom was assumed to be equal for each type of interaction (e.g., the pair and bond interactions are related to two atoms and the atom’s contribution is 1/2, the angle interactions are related to three atoms and so the contribution is 1/3, dihedral interactions related to four atoms and so their contribution is 1/4). Consequently, the sum of potential energy per atom for the whole system will be equal to the total potential energy of the system (Fig. 2b).
b) The average coordination parameter, estimated as the average number of neighbours per atom. Neighbour of an atom is considered any atom that is away less than a cutoff distance. This distance has been defined as 2.4 times the ionic radius if there is only one type of atoms (i.e., one species), 1.2 times the sum of ionic radii of the atom types if there only two atom types/species and 5 Å if more than one atom types exist in the system. The atomic radii are available through the following link (http://abulafia.mt.ic.ac.uk/shannon/radius.php).
c) The common neighbour parameter (CNP), a useful measure of the local crystal structure around an atom and can be used to characterise whether the atom is part of a perfect lattice, a local defect, or at a surface. The formula by Tsuzuki *et al.* [32] can be found in Eq. 3 where *Q* is the common neighbourhood parameter for atom i, ni is the number of nearest neighbors of atom *i*, and *n*_*ij*_ are the common nearest neighbours between atom i and atom *j*, *r*_i_ is the position vector of atom i so that vector $r_{\mathrm{ij}}=r_{j}-r_{i}$. The average value of *Q* parameter is the descriptor that NanoConstruct calculates.
  (3) $$Q=\frac{1}{n_{i}}\sum_{j=1}^{n_{i}}{\left\|\left(\sum_{k=1}^{n_{\mathrm{ij}}}r_{\mathrm{ik}}+r_{\mathrm{jk}}\right\|\right)}^{2}$$
d) The hexatic order parameter (real and imaginary part) can be used as a measure to detect hexagonal symmetry and it is defined by Eq. (4) where N is the number of the nearest neighbours of the *k* atom and *θ* is the angle formed by the bond vector $r_{\mathrm{kj}}=r_{I}-\;r_{k}\;$and x axis. The real part is called “first hex parameter” while the imaginary part is called “second hex parameter” respectively and their average values per atom are used as descriptors.

(4) $$q_{k}=\frac{1}{N}\sum_{j=1}^{N}e^{i6\theta \left(r_{\mathrm{kj}}\right)}$$

Average coordination parameters, common neighbour parameters and hexatic order parameters for other cutoff values are expected to be added in an updated version of NanoConstruct.

### 2.3 Description of NanoConstruct’s graphical user interface and its integration into the platform

#### 2.3.1 Description of NanoConstruct’s graphical user interface and its integration into the Enalos Cloud Platform

**Fig. 3:**
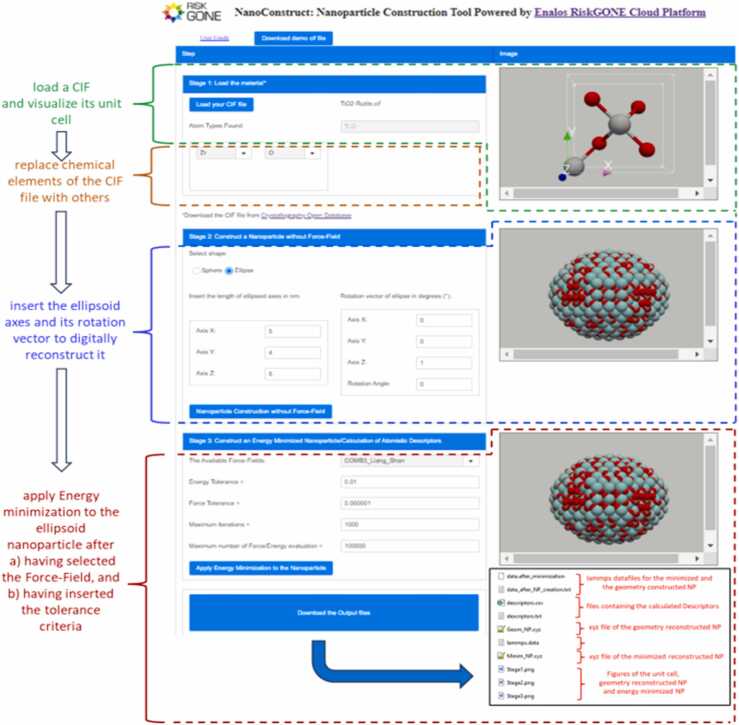
Graphical User interface (GUI) of Nano Construct and its application for the digital reconstruction of ZrO_2_ (rutile-like structure) ellipsoid NP having started from TiO_2_ (rutile) CIF file. The area outlined by the green dashed line illustrates the stage of loading of the CIF file and the visualization of its corresponding unit cell. The area outlined by the orange dashed line shows the dropdown buttons which can be used to replace a chemical element of the unit cell with another, while the area outlined by the blue dashed line illustrates the text button where the user inserts the ellipsoid axes and its rotation vector. The area outlined by the red dashed line illustrates the Force-Field drop-down button and text buttons used to insert the energy minimization tolerance criteria. By the end of the program execution, the user can download files such as the atomistic descriptors (for the whole NP as well as the core and surface layer of 4 Å), the LAMMPS datafiles and XYZ files of the reconstructed NPs. All these files are illustrated in the black box at the bottom of this Figure.

NanoConstruct integrates the aforementioned methodology for the reconstruction of *in silico* NPs and calculation of their descriptors. The GUI is shown in Fig. 3, allowing non-coding experts, i.e., NP designers, product formulators, applications specialists or regulators, to digitally reconstruct the NPs of their choice and calculate their atomistic descriptors. The user can easily select the NP composition (material), diameter for a spherical NP, the ellipsoid axes lengths and the rotation vector for ellipsoidal NPs, and a Force-Field from a pre-selected drop-down list, without needing in-depth knowledge of the background calculations or familiarity with computer programming languages or workflows. The process is divided into three independent steps, as follows.

**Step 1** involves selecting and loading a material using a CIF file from the COD Database which is accessible through a link provided in NanoConstruct’s GUI (see Fig. 3) while users can also use a demo CIF file (Rutile phase of TiO_2_) which can be downloaded from the NanoConstruct’s GUI. Once a CIF file is uploaded, NanoConstruct displays the material’s unit cell and its chemical elements, confirming the selected material for simulation.

**Step 2** allows users to optionally replace a chemical element of the chosen material with another from the same group in the periodic table, facilitating the exploration of new structures. This feature, which limits replacement to adjacent elements in the periodic table to avoid unrealistic configurations, enhances the tool's versatility for researching NP configurations and properties including stability and reactivity.

**Step 3** focuses on defining the NP's shape. Users input parameters for either a spherical or ellipsoidal NP through a straightforward interface, with the platform offering distinct options for each shape. For ellipsoidal NPs, detailed information like the rotation vector and axes lengths is required. This stage culminates in the visual display of the constructed NP, allowing for further adjustments and analysis.

**Step 4** introduces the selection of a force-field and the application of energy minimization to the NP, followed by the generation of an output file including the calculated atomistic descriptors of the NPs.

The geometric construction of a NP by NanoConstruct might not align perfectly with Its actual structure due to the different numbers of neighbouring atoms in the surface and core regions. This variation affects the potential energy, encouraging atoms to move towards a configuration that minimises the system’s total energy. The most probable configuration of a system in thermal equilibrium is the one with the lowest potential energy [30]. After the NP construction, NanoConstruct suggests a default force-field that is less generic and more specific to the NP's chemical elements. Experienced users can select an alternative force-field from a dropdown list, with the OpenKIM database [33] serving as a resource for force-field parameters.

Besides OpenKIM, NanoConstruct includes other force-fields, like the charge-optimised many-body (COMB) potential and continues to update its database with new options. After the selection of a Force-Field, the energy minimization step can be applied [34]. This step, governed by energy tolerance, force tolerance, the number of iterations, and force calculations, stops when any of these criteria are met. The conjugate gradient algorithm which is available in LAMMPS [35] is used for energy minimization, and users are advised to select another force-field if convergence is not achieved.

NanoConstruct is integrated into the Enalos Cloud Platform, a comprehensive suite of predictive models provided as web services, enhancing the utility of NanoConstruct and enabling its integration with other available tools and models, for example by providing NP configurations as inputs for subsequent biomolecule corona determination or for prediction of cellular attachment and toxicity. Developed by NovaMechanics Ltd, the Enalos Cloud Platform is a freely accessible online resource in cheminformatics and nanoinformatics, offering user-friendly GUIs for a suite of nanoinformatics modelling tools. This integration lowers the barriers for conducting complex scientific computations by eliminating the need for programming skills. It supports advanced data analysis and modelling across various scientific fields, including Safe (and Sustainable) by Design (SbD) and regulation, making it accessible to a broader audience.

The Enalos platform excels in combining different data sources, which is crucial for computer-aided drug discovery, materials design, and decision-making processes. Its design, that is intentionally friendly to non-experts in computational approaches, provides access to cutting-edge modelling tools vital for hazard prediction and risk assessment in diverse scientific and industrial settings. By employing the Software as a Service (SaaS) model, it makes computational models like NanoConstruct not just accessible but easy to use. This meets the demands of researchers and scientists for efficient, reliable tools that are simple to navigate, marking a significant advancement in leveraging cloud technology for scientific research and computation. A step-by-step video guide for NanoConstruct can be found online (https://www.youtube.com/watch?v=n5kFS291aQk).

## 3 Results and discussion

### 3.1 Implementation of NanoConstruct to find the most stable shape of a ZrO_2_ NP

To showcase the capabilities of NanoConstruct, a pivotal first step was to determine the minimum energy configuration of a ZrO_2_ NP at a predefined volume. This approach was chosen to highlight how NanoConstruct could be utilised to explore and identify the most stable structures of NPs, which is essential for their practical application in various fields. The CIF file for TiO_2_ rutile with COD ID 1532819 produced by Okrusch *et al.* [36], was selected. Next all the Ti atoms were replaced with Zr ones using the replacement capability offered by NanoConstruct’s GUI.

Next, NanoConstruct was employed to meticulously determine the ellipsoid of the most stable ZrO_2_ rutile-like structured NP, with the volume constraint of 33.51 nm³ . To achieve this objective, the rotation vector (0, 0, 1) and a rotation angle 0 degrees were selected for all cases while the energy minimization parameters applied were: Available Force-Field: Charged Optimised Many-Body 3rd Generation (COMB3) Force-Field [34] as included in LAMMPS version of 23 June 2022 [35], Energy Tolerance: 0.0001, Force Tolerance: 10^−6^, Maximum Iterations 1000 and Maximum number of Force/Energy Evaluation: 10^5^ (see the paper describing the ASCOT web application for more information about these parameters [29]). For each ellipsoid, the average potential energy per atom was calculated, establishing a clear metric for stability assessment.

The comprehensive data set revealed that the ellipsoid with Run ID 23 (see Table 1), with length of ellipsoid axes *D*_a_ = 5.00 nm, *D*_b_ = 3.90 nm, *D*_c_ = 3.28 nm, and maintaining the volume at 33.51 nm³ , appeared to be the most stable with the smallest average potential energy per atom, recorded at − 7.011 eV. This is a strong indication that the most preferable crystal growth axis and the ellipsoidal shape of ZrO_2_ rutile structured NPs. Ellipsoid TiO_2_ rutile NPs (also having tetragonal space group) were found to be stable in the literature too [37].

**Table 1:** Length of Ellipsoid axes (i.e., ellipsoid diameters) at x, y and z principal directions. The Run ID 23 (bold values) illustrates the most stable ellipsoid. The rotation vector (0, 0, 1) and a rotation angle 0 degrees was selected for all cases while the energy minimization parameters were: Available Force-Field: Charged Optimised Many-Body 3rd Generation (COMB3) Force-Field [34] as included in LAMMPS version of 23 June 2022 [35], Energy Tolerance: 0.0001, Force Tolerance: 10^−6^, Maximum Iterations 1000 and Maximum number of Force/Energy Evaluation: 10^5^ (see the paper describing the ASCOT web application for more information about these parameters [29]).

***Run ID***	***D***_**x**_**(nm)**	***D***_**y**_**(nm)**	***D***_**z**_**(nm)**	***V*****(nm**^**3**^**)**	***Avg Potential Energy per Atom*****(eV)**
1	4.00	4.00	4.00	33.51	-6.721
2	4.00	3.00	5.33	33.51	-6.786
3	4.00	2.00	8.00	33.51	-6.824
4	3.00	4.00	5.33	33.51	-6.905
5	2.00	4.00	8.00	33.51	-6.822
6	3.50	3.00	6.10	33.51	-6.838
7	3.00	3.50	6.10	33.51	-6.837
8	3.00	5.00	4.27	33.51	-6.784
9	3.00	4.50	4.74	33.51	-6.972
10	3.00	4.30	4.96	33.51	-6.787
11	3.00	4.80	4.44	33.51	-6.793
12	3.00	4.40	4.85	33.51	-6.951
13	4.00	5.00	3.20	33.51	-6.998
14	4.00	5.20	3.08	33.51	-6.953
15	4.00	4.80	3.33	33.51	-6.795
16	4.00	2.50	6.40	33.51	-6.675
17	4.00	3.50	4.57	33.51	-6.793
18	5.00	4.00	3.20	33.51	-6.997
19	5.00	4.50	2.84	33.51	-6.718
20	5.00	3.50	3.66	33.51	-6.960
21	5.00	3.70	3.46	33.51	-6.752
22	5.00	3.30	3.88	33.51	-6.761
**23**	**5.00**	**3.90**	**3.28**	**33.51**	**-7.011**
24	5.00	3.80	3.37	33.51	-6.992
25	4.50	4.00	3.56	33.51	-6.766
26	4.50	3.00	4.74	33.51	-6.821
27	4.50	2.00	7.11	33.51	-6.852
28	3.00	2.00	10.67	33.51	-6.789
29	5.00	5.00	2.56	33.51	-6.640
30	2.00	3.00	10.67	33.51	-6.810
31	2.00	4.00	8.00	33.51	-6.822
32	2.00	5.00	6.40	33.51	-6.754
33	2.00	2.50	12.80	33.51	-6.683

Taking a closer look at the data from Table 1 it can be concluded that the configurations with more elongated shapes (higher disparity between the dimensions of the principal axes) tend to have higher potential energy values, as seen with Run IDs 3, 28, and 33. This trend suggests that more extreme ellipsoidal shapes may be less stable compared to more compact or balanced ellipsoids. On the other hand, configurations that closely approach more symmetrical shapes, such as *Run ID* 23, tend to exhibit lower potential energy, indicating greater stability.

**Fig. 4:**
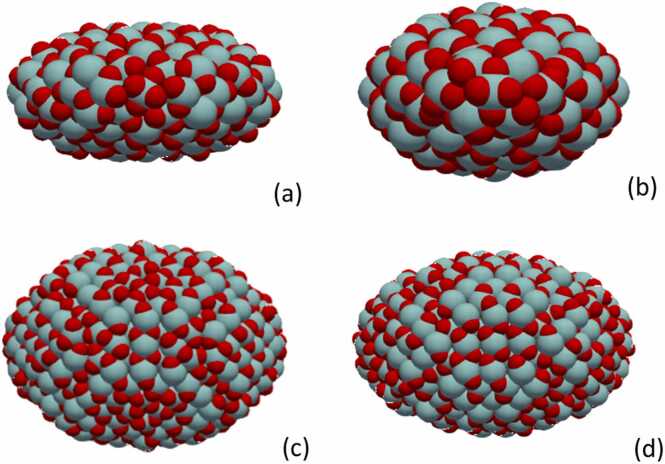
Illustration of four ZrO_2_ NP configurations generated by NanoConstruct. (a) Run ID 3 with elongated shape and high potential energy, (b) Run ID 28 with an even more extended structure, also with high potential energy, (c) Run ID 18 representing a more compact form with reduced potential energy, (d) Run ID 22, another compact shape, close to the lowest energy configuration found in the study. Each configuration is visualised to highlight the impact of NP dimensions on stability, as indicated by their average potential energy per atom. Zr atoms are depicted in light blue and O atoms in red.

To provide a visual demonstration of the differences observed Table 1, Fig. 4 showcases four distinct configurations of ZrO_2_ NPs. This visual representation is critical in highlighting the relationship between the physical structure of the NPs and their stability, as suggested by their average potential energy per atom. The Zr and O atoms are depicted with light blue and red colours respectively. In Fig. 4, configuration (a) corresponds to Run ID 3, which shows an elongated ellipsoidal shape. This NP has a visibly extended structure along one axis, which, as indicated in Table 1, corresponds to a high potential energy and suggests lower stability. Similarly, configuration (b) representing Run ID 28, possesses an even more pronounced elongation, which correlates with an increase in potential energy, implying that such stretched forms may be less stable. On the contrary, configurations (c) and (d), corresponding to Run IDs 18 and 22 respectively, illustrate more compact and symmetrical shapes. These NPs demonstrate how a reduction in elongation—a closer approach to an equiaxed form—appears to contribute to lower potential energy and thus higher stability. The compactness of these structures is readily apparent when compared to the more elongated shapes of (a) and (b).

To ensure that the constructed NP is not in a local minimum instead of a global one, molecular dynamics (MD) simulations at elevated temperatures are recommended, which will facilitate the system in surmounting potential energy barriers, thus reaching finally the global minimum. Because of time limitations of a web application, these simulations are beyond the scope of NanoConstruct. However, the LAMMPS datafiles generated as output by NanoConstruct could be used as input in LAMMPS [35] by the user to conduct these MD simulations independently. Furthermore, comparing the potential energy values across different configurations reveals a nuanced landscape of stability for ZrO_2_ NPs. For instance, configurations with one significantly longer axis (e.g., Run IDs 3 and 28) showcase a potential energy range that hints at less stability, likely due to increased surface energy associated with more elongated shapes.

The configuration with the lowest potential energy (Run ID 23) not only highlights the capability of NanoConstruct to identify stable NP shapes but also underscores the importance of controlling NP dimensions for specific applications by trying to eliminate the crystal growth in a specific direction. The findings from Table 1 provide crucial insights into the relationship between shape and stability, which could be instrumental in designing NPs for catalysis, drug delivery, and other nanotechnology applications where stability and reactivity are key.

### 3.2 Application of NanoConstruct to find the pathways of crystal growth of a ZrO_2_ NP

**Fig. 5:**
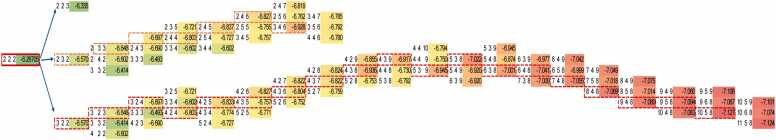
Energy Pathway Map for Crystal Growth of Ellipsoidal ZrO_2_ NPs. This map plots the path of NP growth, beginning with a spherical base of 2 nm diameter and transitioning through a series of ellipsoidal transformations. Each ellipsoid's lengths of axes and corresponding average potential energy per atom are systematically displayed, with colour coding to indicate stability levels—red for configurations of higher stability and green to yellow for lower stability. The pathway concludes with the most stable identified structure at [11 5 8] nm ellipsoid axis length, showcasing an average potential energy of − 7.124 eV. The rotation vector (0, 0, 1) and a rotation angle of 0 degrees was selected for all the cases using the aforementioned energy minimization parameters.

According to classical nucleation theory [38], the unfavourable interface contribution to the free energy is dominant at the early stages but becomes favourable after a critical radius (if the NP is spherical) or ellipsoidal principal axis radii (if the NP is ellipsoidal) is reached. NanoConstruct can also be used for identification of the exact crystal growth route by applying the procedure of the methodology section. During this procedure, different ellipsoids that can be created after the addition of atoms to the surface of the NP are investigated according to the average potential energy per atom and the route with the lowest average potential energy per atom is selected to be the most probable for the crystal growth of the NP. Because of the large number of possible ellipsoids, the routes of Fig. 5 were investigated in this paper. Fig. 5 serves as a detailed flow diagram or pathway map that systematically delineates a series of ellipsoidal NPs, each characterised by distinct dimensions and documented by their respective average potential energy per atom, measured in electron volts (eV). The journey through this energy landscape commences with a spherical NP with a diameter of 2 nm, signified by the initial ellipsoidal axes length of 2 nm per direction (denoted as [2 2 2] for convenience). This notation is also used to describe an ellipsoid: as the map unfolds, we observe a meticulous exploration of alternate routes—each route representing a transformation of the NP's ellipsoidal dimensions, accompanied by specific rotations characterised by vector components and angular values.

In this detailed map, each ellipsoid is accompanied by an energy value, where the convention is such that a lower energy value is indicative of a more stable ellipsoidal configuration. In Fig. 5a colour coding is followed to visually discriminate between varying levels of stability The colour scheme employed is that red is used to denote configurations of higher stability, while green and yellow signify configurations of lower stability.

The chosen trajectory for crystal growth is marked by a sequence of ellipsoids with gradually lower energy values, culminating in an ellipsoid defined by the lengths of ellipsoid axes equal to [11 5 8], which possesses an average potential energy of − 7.124 eV. This configuration is noted as the most energetically favourable structure identified through this analytical approach. The progression along this chosen path is demonstrative of a systematic and methodical selection process based on potential energy considerations, aimed at discovering the optimal structural configuration for NP growth.

Taking a closer look at the trends in crystal growth as presented in Fig. 5, the following conclusions can be drawn. Firstly, there is a notable trend where configurations with certain proportions between axes lengths (*D*_*x*_, *D*_*y*_, and *D*_*z*_) tend to have lower potential energies, indicating higher stability. For example, configurations where the ellipsoid axis length *D*_*y*_ is between 3 to 5 nm and the ellipsoid axis length *D*_z_ is 8 nm seem to be more stable with lower potential energy values (e.g., [6 3 8] at −7.001 eV and [5 3 8] at −7.022 eV). Moreover, NPs with more symmetrical dimensions (where one axis isn't significantly longer or shorter than the others) generally have higher potential energies, suggesting less stability. Conversely, a moderate asymmetry tends to correlate with lower energy and higher stability, as seen in the most stable configuration of [11 5 8] with an energy of − 7.124 eV.

Then, as the NP grows larger (ellipsoid axis a is increased from 5 to 11 nm), there appears to be a decrease in energy, suggesting increasing stability. This implies that there could be a critical size or range where the NP formation becomes energetically favourable. The sequence of NP configurations suggests an optimal growth path that minimises potential energy as well as plateaus in the potential energy as the ellipsoid axis’s lengths increase beyond a certain point, which may represent reaching a stable crystalline structure that doesn't significantly change with further growth in size.

The significance of this map extends beyond mere representation; it offers a conceptual framework to hypothesise the physical processes underlying the crystal growth of NPs. The conditions modelled by the NanoConstruct tool serve as a proxy for real-world phenomena, allowing for the projection of these computational findings to tangible experimental scenarios. In essence, the elucidation of this energy pathway provides valuable insights into the theoretical growth patterns that NPs may exhibit, laying a robust foundation for further empirical investigation and potential technological applications.

**Fig. 6:**
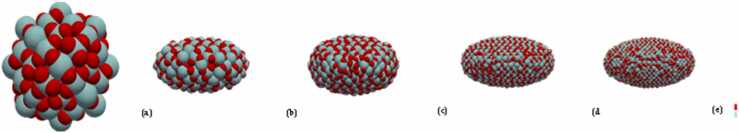
Evolution of ellipsoidal ZrO_2_ NP configurations during crystal growth as predicted by the NanoConstruct computational model. (a) Initial near-spherical shape with lengths of ellipsoid axes equal to [2 2 2] nm representing the nucleation phase. (b) Early growth stage with elongated shape along one axis, with lengths of ellipsoid axes equal to [4 2 4] nm. (c) Intermediate growth stage, further elongation and asymmetry observed, with lengths of ellipsoid axes equal to [5 3 9] nm. (d) Advanced growth stage approaching stability, with lengths of ellipsoid axes equal to [8 4 8] nm. (e) Near-final growth stage with lengths of ellipsoid axes equal to [10 5 8] nm, indicative of a shape with minimised surface energy and increased stability as per the simulation outcomes. Each configuration is color-coded to distinguish between Zr (light blue) and O (red) atoms.

Fig. 6 captures the sequential growth stages of an ellipsoidal ZrO_2_ NP as predicted by the computational simulations. Starting with image (a), representing the early nucleation stage, the NP exhibits a nearly spherical shape with the lengths of the ellipsoid axes equal to [2 2 2] nm. As the crystal growth progresses to image (b), with length of ellipsoid axes equal to [4 2 4] nm, we observe an elongation that suggests a shift towards anisotropic growth, likely influenced by minimization of the NP’s surface energy. Moving to image (c), where the lengths of the ellipsoid axes are equal to [5 3 9] nm, the elongation becomes more pronounced, possibly indicating the preferred growth direction or facets of the crystalline structure. Image (d) shows further advancement in growth with the lengths of the ellipsoid axes equal to [8 4 8] nm, where the NP begins to stabilise into an ellipsoidal shape that likely approaches a thermodynamically favourable configuration. Finally, image (e) presents a near-final growth stage with the lengths of the ellipsoid axes equal to [10 5 8] nm, suggesting a stable ellipsoidal configuration that could represent the global energy minimum for the given size. The Zr and O atoms are color-coded in light blue and red, respectively, allowing for visual differentiation and providing insight into the crystal structure's evolution at the atomic level.

By illustrating the NP’s evolution, Fig. 6 serves as a bridge between theoretical computational models and potential real-world synthesis outcomes, offering a visual aid that enhances the reader's comprehension of the complex processes discussed in the paper. In the meticulous quest to ascertain the most stable configuration of an ellipsoidal NP with lengths of ellipsoid axes equal to [10 5 8] nm, a thorough investigation was conducted focusing on the influence of rotational angles on the system's energy. The empirical exploration spanned the full spectrum of angles from 10 to 180 degrees, methodically evaluating the NP's potential energy at each juncture. Despite this comprehensive analysis, the results illustrated a negligible variation in stabilization energy, with the initial rotational angle of 0 degrees yielding an energy of − 7.127 eV, which stands as the benchmark of stability. The results can be found in Table 2 and are in good agreement with the cohesive energy and structure properties mentioned in literature [39], [40], [41], [42] for ZrO_2_.

**Table 2:** Ellipsoid diameters at x, y and z principal directions for different rotation angles. The rotation vector (0, 0, 1) was selected for all cases, while the energy minimization parameters used were: Available Force-Field: Charged Optimised Many-Body 3rd Generation (COMB3) Force-Field: Charged Optimised Many-Body 3rd Generation (COMB3) Force-Field [34] as included in LAMMPS version of 23 June 2022 [35], Energy Tolerance: 0.0001, Force Tolerance: 10^−6^, Maximum Iterations 1000 and Maximum number of Force/Energy Evaluation: 10^5^ (see the paper describing the ASCOT web application for more information about these parameters [29]).

***rotation angle*****(**^**o**^**)**	***D***_**x**_**(nm)**	***D***_**y**_**(nm)**	***D***_**z**_**(nm)**	***Avg Potential Energy per Atom*****(eV)**
0	10	5	8	-7.127
10	10	5	8	-7.124
20	10	5	8	-7.081
30	10	5	8	-7.059
40	10	5	8	-7.039
50	10	5	8	-7.028
60	10	5	8	-7.050
70	10	5	8	-7.050
80	10	5	8	-7.078
90	10	5	8	-7.003
100	10	5	8	-7.123
110	10	5	8	-7.085
120	10	5	8	-7.075
130	10	5	8	-7.039
140	10	5	8	-7.027
150	10	5	8	-6.996
160	10	5	8	-7.040
170	10	5	8	-7.076
180	10	5	8	-7.122

As the rotation angle increased, no notable enhancement in stability was observed. The energy values fluctuated within a narrow band, with the maximum recorded energy being − 6.996 eV at a 150-degree rotation and the minimum at the starting point. This indicates a plateau in the energy landscape; the system appears relatively indifferent to the rotational adjustments within the tested range suggesting that the NP's configuration remains robust against rotational distortions. This outcome reinforces the understanding that the NP possesses an intrinsic structural resilience, with its lowest energy configuration at a rotation angle of 0 degrees remaining the benchmark of stability within the parameters of this study.

## 4 Discussion

A different methodology for the prediction of the shape of particles has already been proposed by Wulff [43]. The Wulff construction of a particle requires the calculation of the surface tensions for an infinite number of *hkl* planes. Due to the limited experimental data for the surface tension of solids for both their interface with vacuum [44] and their interface with other fluids, the surface tension calculations are often based on density functional theory [45] and are time-consuming. This leads to the calculation of a limited number of *hkl* planes of the bulk material so that the Wulff construction methodology is feasible. Consequently, the Wulff approach ignores the planes with high *hkl* which may lead to the construction of unrealistic nanoparticles. While the Wulff approach is expected to be true for large particles due to its assumptions (macroscopic treatment of the structure), nanoparticles structures differ from the bulk structures which enable them to manifest different properties than those of the bulk material and underpins their use for many applications. The Wulff construction does not consider the edge and vertex energies in its calculations, which are expected to be significant for small nanoparticles and less important for larger sized particles. For example, size-dependent shapes have been identified for platinum nanoparticles [46].

Concerning the crystal growth process, the Wulff construction methodology cannot be used directly as it predicts only the selected facets which are flat while atoms are deposited onto the surface gradually to make a new layer of the facet. This gradual deposition of atoms may not be favourable due to the absence of the neighbouring layer atoms, while for faces with higher surface tensions it becomes more favourable. A similar deposition approach is included in the crystal growth process described in the current work.

As we have shown previously (see Figure 4 of Ref 29), by selecting the construction of an ellipsoidal nanoparticle, the constructed nanoparticle is actually inscribed to the ellipsoid polyhedron which consists of different *hkl* planes. The selection of different ellipsoids (i.e., ellipsoid lengths and orientation angles) leads to different *hkl* planes. If the surface tension of all *hkl* planes is not negligible, the ellipsoid approach is expected to be nearest to the real nanoparticle structure than the Wulff construction which is better when the surface tension (in absolute value) is important only in few *hkl* directions. Because TiO_2_ (rutile) nanoparticles have been found to be ellipsoidal [47], the strategy of ellipsoidal nanoparticles was mentioned for similarly structured materials. A new tool that will apply the Wulff methodology is already under construction and it is intended to be used in conjunction with NanoConstruct to investigate more precisely the crystal growth of materials. The shape of the particles is often known through Scanning Electron Microscopy Images. Where these images are available in advance, one can select which approach (between NanoConstruct and Wulff) is better. Finally, the size of the nanoparticle is not considered as a parameter in the Wulff construction, due to the use of the surface tension of bulk materials despite the fact that surface tension depends on the nanoparticle size, whereas nanoparticle size is a tuneable parameter in NanoConstruct.

## 5 Conclusion

In this paper, we presented the digital construction and optimization of ellipsoidal ZrO_2_ rutile-like structured NPs using the NanoConstruct toolbox. This computational approach has provided significant insights into the most stable configurations of ZrO_2_ NPs, which is crucial for their application across various domains, including medicine, electronics, and materials science. Our methodology for generating *in silico* NPs is structured around the digital reconstruction of the unit cell, formation of NPs of the target shape (ellipsoid in the example shown herein), and optimization processes, followed by calculation of a panel of atomic descriptors of the NPs (both core and surface atoms) enabled a comprehensive analysis of the structural and physicochemical characteristics of ZrO_2_ NPs. The findings underscore the vital role of shape and geometrical optimization in determining NP stability, emphasizing the significance of computational tools in nanotechnology for designing NPs with tailored properties for specific applications.

The integration of the NanoConstruct toolbox into the Enalos Cloud Platform has demonstrated the utility of computational tools in making complex simulations accessible, thus advancing nanotechnology research and material design. Through the detailed analysis of ZrO_2_ NPs, we have identified configurations that contribute to understanding NP stability, emphasizing the impact of geometric and atomistic descriptors in predicting NP behaviour. This work highlights the capabilities of the NanoConstruct toolbox and underlines the importance of digital construction and analysis in the field of nanotechnology, paving the way for novel material creation and fully *in silico* nanosafety assessment.

Our research provides a pathway towards the design of NPs with specific properties, enhancing their application in various fields. By offering insights into the most stable configurations of ellipsoidal NPs and illustrating the significance of computational analysis in nanotechnology, this study contributes to the broader understanding of NP stability and design, fostering innovation in the creation of materials with optimised properties for diverse applications.

## Funding

This research was funded by the European Union’s Horizon 2020 Research and Innovation Programme via the RiskGone project (grant agreement nº 814425) and the European Union’s H2020 Marie Skłodowska-Curie Actions via CompSafeNano under (grant agreement nº 101008099).

## CRediT authorship contribution statement

Conceptualization, P.K.; methodology, P.K., E.V; software, P.K., N.S., A.T.; validation, D.Z. and I.L.; formal analysis, P.K.; investigation, D.Z.; writing—original draft preparation, D.Z. and P.K.; writing—review and editing, G.M., K.T., A.A., A.T., N.V. and I.L.; visualization, D.Z. and P.K.; supervision, K.T., I.L. and A.A.; project administration, A.A.; funding acquisition, K.T., I.L. and A.A.

## Declaration of Competing Interest

PK, DZ, NS, AT and AA are employed by NovaMechanics a cheminformatics and materials informatics company.
